# Association between serum calcium and in-hospital mortality in patients with severe fever with thrombocytopenia syndrome: a retrospective cohort study

**DOI:** 10.1186/s12985-026-03232-z

**Published:** 2026-07-07

**Authors:** Huan Wang, Sisi Fang, Hua Wang, Xin Zheng

**Affiliations:** 1 Department of Infectious Diseases, Union Hospital, Tongji Medical College, Huazhong University of Science and Technology, Wuhan, 430022 China; 2 School of Mathematics and Physics, China University of Geosciences, Wuhan, 430074 China

**Keywords:** Severe fever with thrombocytopenia syndrome, Serum calcium, Mortality

## Abstract

**Objective:**

Severe fever with thrombocytopenia syndrome (SFTS) is a viral infectious disease characterized by high mortality. Calcium dysregulation is implicated in viral replication and host immune response. We aimed to evaluate the association between serum calcium levels and in-hospital mortality in patients with SFTS.

**Methods:**

In this retrospective, single-centre cohort study, we enrolled 809 laboratory-confirmed SFTS patients admitted between April 2017 and June 2024. Hypocalcemia was defined as corrected calcium < 8.12 mg/dL. Multivariate logistic regression, restricted cubic spline (RCS), and longitudinal analyses were employed to assess associations between serum calcium and mortality.

**Results:**

The in-hospital mortality rate was 11.87% (96/809). Hypocalcemia was observed in 72.68% of patients (588/809) with SFTS upon admission. Multivariate logistic regression analysis revealed that lower serum calcium was an independent predictor of in-hospital mortality, alongside older age, neurological symptoms, higher viral loads, lower albumin levels, elevated creatinine, and prolonged activated partial thromboplastin time (APTT). Restricted cubic spline (RCS) analysis suggested a visual L-shaped pattern between serum calcium levels and in-hospital mortality, but without statistically significant evidence of non-linearity. Dynamic analysis showed that serum calcium levels increased and normalized by day 6 in survivors but peaked and declined thereafter in non-survivors. Hypocalcemia was independently associated with hypoalbuminemia, hypoglobulinemia, hyponatremia, and monocytopenia. Furthermore, serum calcium levels were inversely correlated with viral load, creatine kinase, APTT, IL-6, and IL-10 (all *p* < 0.05).

**Conclusions:**

Hypocalcemia is exceedingly common in SFTS patients and serves as a robust, independent prognostic marker for in-hospital mortality. Its association with high viral replication and host inflammatory response underscores its clinical significance. Serial monitoring of serum calcium may serve as a useful marker for risk stratification in SFTS.

**Supplementary Information:**

The online version contains supplementary material available at https://doi.org/10.1186/s12985-026-03232-z.

## Introduction

SFTS, an emerging tick-borne infectious disease caused by the SFTS virus (SFTSV), has been officially named *Dabie bandavirus* (DBV), which belongs to the *Bunyavirales order*, *Phenuiviridae family*, and *Bandavirus genus* [1]. SFTS was first identified in China in 2009 and rapidly spread in South Korea, Japan, the USA, Vietnam, Myanmar and Taiwan [2–7]. It is characterized by hemorrhagic fever, thrombocytopenia, leukocytopenia, neurological complications, electrolyte imbalances, multiple organ failure, and eventually death, with a fatality rate of 12%-50% [8]. Due to its high mortality rate and the possibility of an outbreak of as a global pandemic, SFTS was declared one of the priority infectious diseases by the WHO in 2018 [9]. Ribavirin, favipiravir (T-705), and calcium channel blockers have been previously reported for their antiviral effects against SFTSV [8, 10, 11]. Nonetheless, to date, no effective vaccines or antiviral agents have been approved for the treatment of SFTSV infection. Hence, identifying the risk factors associated with mortality and taking early action are crucial to reducing the death rate among SFTS patients.

Calcium is a vital element that plays a critical role in various physiological functions. It is crucial for maintaining proper cardiac function, facilitating muscle contractions, ensuring the structural integrity of bones, and serving as a signaling molecule in various biochemical pathways. Calcium ions (Ca^2+^) act as second messengers within mammalian cells involved in the regulation of almost all cellular processes and play a fundamental role in viral replication mechanisms, including Middle East Respiratory Syndrome Coronavirus (MERS-CoV), COVID-19, Ebola viruses, Dengue fever, hepatitis B virus, and SFTSV [11–16]. Previous study by Li et al. revealed that calcium channel blocker benidipine hydrochloride inhibit SFTSV infection through reducing cellular Ca^2+^ uptake [11]. Thus, alterations in calcium homeostasis might play a role in the disease severity and mortality in patients with SFTS.

Hypocalcemia has been identified as a risk factor associated with increased mortality in patients with COVID-19, Ebola, Dengue fever and SFTS [11, 17–19]. Previous studies indicated that the incidence of hypocalcemia in COVID-19 patients range from 59.3% to 76.6% and found that total calcium ≤ 2.02 mmol/L or 8.38 mg/dL had increased risk of in-hospital mortality [17, 20, 21]. Several studies found that hypocalcemia was also associated with increased mortality in non-viral infectious diseases such as acute pulmonary thromboembolism, heart failure and chronic kidney disease [22, 23]. The mechanisms underlying this may involve pro-inflammatory cytokine release, end-organ resistance to parathyroid hormone, vitamin D deficiency or insufficiency [24]. To the best of our knowledge, there have been very few studies investigating serum calcium levels and the risk of death in SFTS patients [11]. However, these two studies only investigated the impact of serum calcium on prognosis without considering the influence of albumin on serum calcium levels. Moreover, they did not provide the optimal critical value of serum calcium for estimating the disease severity or data on the dynamic changes in serum calcium levels during hospitalization.

Our study aimed to investigate the prevalence of hypocalcemia in SFTS patients upon hospital admission, evaluate the association between serum calcium levels and clinical outcomes, analyze dynamic changes in serum calcium levels during hospitalization, and determine the optimal critical threshold of serum calcium for assessing disease severity. These findings may facilitate early identification of high-risk patients.

## Patients and methods

### Patients and data collection

A total of 816 laboratory-confirmed SFTS patients admitted to the Department of Infectious Diseases at Wuhan Union Hospital from April 2017 to June 2024 were retrospectively analyzed. Laboratory confirmation of SFTS required meeting one or more of the following criteria: (a) detection of SFTSV RNA in the patient’s serum; (b) detection of IgM antibodies against SFTSV or a four-fold increase in serum IgG antibodies during the convalescent phase; or (c) detection of the SFTSV genome via next-generation sequencing. After excluding 7 patients with missing serum calcium records, 809 patients were ultimately included in the study. Patients were stratified into three groups based on serum calcium levels: G1 <7.0 mg/dL (*n* = 59), 7.0 ≤ G2<8.12 (*n* = 529), G3 ≥ 8.12 (*n* = 221) (see S1 Fig). Two trained physicians retrospectively collected data on demographics, underlying diseases, clinical characteristics, laboratory findings, comorbidities, and overall prognosis from medical records. As this was a retrospective, single-centre study, the findings may not be generalisable to other populations or healthcare settings.

### Statistical analysis

All statistical analyses were conducted using R (version 4.4.0; R Foundation for Statistical Computing, Vienna, Austria). Continuous variables were presented as median (1st quartile, 3rd quartile) or mean (± standard deviation). Significant differences between two groups were assessed using the Student’s t-test or the nonparametric two-tailed Mann-Whitney U test. Categorical variables were expressed as percentages (%), and significant differences between two groups were analyzed using the chi-square test or Fisher’s exact test. Differences among multiple groups were determined using one-way analysis of variance (ANOVA) or the Kruskal-Wallis test. Multivariate logistic regression analyses were used to identify independent risk factors for mortality and corrected calcium levels. Variables for multivariable logistic regression models were selected based on clinical relevance and statistical significance in univariate analysis (*p* < 0.05). To avoid model overfitting, we ensured that the number of events per variable (EPV) exceeded 10 (96 in-hospital deaths with 7 variables included). To address potential multicollinearity, variance inflation factor (VIF) was calculated for each variable, and those with VIF > 5 were excluded. Missing laboratory data were handled using multiple imputation with predictive mean matching. Missing data occurred only in secondary laboratory markers (0%–25.22%), with core variables fully observed. The data were judged missing at random (MAR) based on baseline comparisons and clinical logic. A complete-case sensitivity analysis confirmed the robustness of the multiple imputation results. Survival analysis, stratified by corrected calcium levels across the four groups, was performed using Kaplan-Meier (KM) curves with the log-rank test. Restricted cubic splines (RCS) were used to further investigate the association between calcium levels and SFTS mortality. The dynamic changes in serum calcium levels, tracked from admission to discharge or death, were visualized using locally weighted scatterplot smoothing (LOESS). The relationships between serum calcium levels and viral load, creatine kinase, and APTT, IL-6, IL-10, CD4^+^T cell at hospital admission were assessed using linear regression models to evaluate the strength and significance of these associations. All statistical tests were two-tailed, and a p-value < 0.05 was considered statistically significant.

## Results

### Baseline characteristics of the study population

As shown in Table 1, a total of 809 patients were enrolled in this study, of whom 96 (11.87%) died during hospitalization. The median age of the study population was 63 years (interquartile range [IQR]: 55–70), with 342 (42.27%) male participants. Major comorbidities included hypertension in 191 patients (23.61%), diabetes mellitus in 93 (11.50%), chronic obstructive pulmonary disease (COPD) in 33 (4.08%), and chronic hepatitis B (CHB) in 83 (10.26%). Neurological and hemorrhagic manifestations occurred in 26.70% and 12.48% of cases, respectively. The median hospital stay was 9 days (interquartile range [IQR]: 7–12), with 21 cases (2.60%) requiring intensive care unit (ICU) transfer. Patients were stratified into survival (*n* = 713) and non-survival (*n* = 96) groups based on clinical outcomes. Comparative analysis revealed significant between-group differences, non-survivors were older than survivors (median age 70 [IQR 62–75] vs. 62 [IQR 54–69] years; *P* < 0.001) and had higher hypertension prevalence (40.61% [39/96] vs. 21.32% [152/713]; *P* < 0.001). No intergroup differences were detected in sex distribution (*P* = 0.472), diabetes mellitus (*P* = 0.356), chronic obstructive pulmonary disease (COPD; *P* = 0.621), or chronic hepatitis B (CHB; *P* = 0.843) prevalence. Notably, the non-survival group demonstrated significantly higher frequencies of neurological manifestations (67.71% vs. 21.18%, *P* < 0.001), hemorrhagic manifestations (20.83% vs. 11.36%, *P* = 0.008), and ICU transfer (9.38% vs. 1.68%, *P* < 0.001).

**Table 1:** Baseline Characteristics and Laboratory Parameters in Patients With SFTS

Variables	Total (*n* = 809)	Survivors (*n* = 713)	Non-survivors (*n* = 96)	*P*
Age (y)	63.00 (55.00, 70.00)	62.00 (54.00, 69.00)	70.00 (63.00, 73.25)	< 0.0001
Sex, male (%)	342 (42.27%)	301 (42.22%)	41 (42.71%)	0.927
**Comorbidity**				
Hypertension	191 (23.61%)	152 (21.32%)	39 (40.61%)	< 0.0001
Diabetes	93 (11.50%)	81 (11.36%)	12 (12.50%)	0.7424
COPD	33 (4.08%)	28 (3.93%)	5 (5.21%)	0.7482
CHB	83 (10.26%)	78 (10.94%)	5 (5.21%)	0.0823
**Complications**				
Neurological symptoms	216 (26.70%)	151 (21.18%)	65 (67.71%)	< 0.0001
Hemorrhagic signs	101 (12.48%)	81 (11.36%)	20 (20.83%)	0.0084
**Laboratory parameters**				
Calcium	7.80 (7.48, 8.16)	7.84 (7.52, 8.16)	7.48 (7.00, 7.82)	< 0.0001
Albumin	31.74 ± 4.48	31.91 ± 4.45	30.41 ± 4.48	0.002
Corrected Calcium	8.45 (8.17, 8.76)	8.48 (8.20, 8.78)	8.22 (7.81, 8.56)	< 0.0001
Viral load (Log10)	3.07(2.06, 4.11)	2.91(2.00, 3.80)	5.07 (4.11, 5.72)	< 0.0001
WBC	2.85 (1.76, 4.78)	2.90 (1.78, 4.90)	2.39 (1.68, 4.04)	0.1302
RBC	4.20 (3.82, 4.60)	4.20 (3.82, 4.61)	4.22 (3.89, 4.54)	0.6886
Hb	126.00(115.00, 139.00)	125.00(115.00, 139.00)	127.50 (116.00, 139.00)	0.4417
PLT	50.00 (34.00, 64.00)	51.00 (36.00, 66.00)	35.50 (24.00, 49.25)	< 0.0001
Lymphocyte	0.69 (0.44, 1.22)	0.70 (0.45, 1.26)	0.57 (0.33, 0.96)	0.0032
Monocyte	0.16 (0.08, 0.37)	0.18 (0.08, 0.39)	0.095 (0.058, 0.173)	< 0.0001
Neutrophil	1.64 (0.95, 3.17)	1.69 (0.92, 3.26)	1.51 (1.08, 2.77)	0.907
ALT	80.00(48.00, 142.00)	77.00 (47.00, 129.00)	118.50 (62.75, 195.75)	0.0003
AST	185.00 (101.00, 362.00)	174.0000 (95.00, 321.00)	409.00 (222.25, 690.50)	< 0.0001
Tbil	9.40 (7.00, 12.80)	9.40 (6.90, 12.80)	9.95 (7.18, 13.05)	0.2994
TP	57.40 (53.30, 61.30)	57.50 (53.40, 61.40)	56.95 (52.15, 60.93)	0.4013
Globulin	25.30 (22.80, 28.40)	25.20 (22.60, 28.20)	25.8500 (23.98, 29.90)	0.0168
Creatinine	70.00 (58.00, 85.20)	68.60 (57.20, 82.48)	90.2500 (70.10, 148.03)	< 0.0001
BUN	4.82 (3.45, 6.58)	4.6200 (3.34, 6.20)	7.56 (5.53, 11.04)	< 0.0001
LDH(Log10)	2.88 (2.69, 3.09)	2.86 (2.67, 3.05)	3.13 (2.90, 3.35)	< 0.0001
CK(Log10)	2.70 (2.36, 3.11)	2.67 (2.33, 3.07)	3.08(2.63, 3.32)	< 0.0001
cTnI	106.00 (44.10, 555.80)	97.70 (40.30, 447.90)	227.10 (92.23, 688.82)	< 0.0001
CKMB	4.70 (1.90, 7.03)	4.50 (1.80, 6.74)	6.28 (3.28, 9.28)	< 0.0001
Sodium	135.60 (133.00, 138.20)	135.60 (133.10, 138.20)	135.40 (132.25, 138.50)	0.4116
Potassium	3.63 (3.30, 3.94)	3.60 (3.29, 3.91)	3.96 (3.53, 4.22)	< 0.0001
Magnesium	0.78 (0.68, 0.86)	0.78 (0.69, 0.86)	0.75 (0.68, 0.83)	0.1325
Phosphorus	0.90 (0.76, 1.08)	0.90 (0.76, 1.07)	0.95 (0.79, 1.17)	0.0206
APTT	50.20 (42.70, 59.80)	48.90 (41.70, 57.40)	67.60 (55.30, 83.83)	< 0.0001
D-dimer	2.90 (1.40, 5.09)	2.57 (1.31, 4.75)	5.01 (2.98, 10.32)	< 0.0001
FIB	2.49 (2.05, 2.97)	2.50 (2.08, 3.02)	2.41 (1.97, 2.78)	0.07
INR	1.03 (0.94, 1.15)	1.02 (0.94, 1.14)	1.09 (1.00, 1.22)	0.0006
PT	13.20 (12.40, 14.40)	13.10 (12.30, 14.30)	13.70 (13.00, 15.03)	< 0.0001
TT	24.40 (20.40, 31.30)	23.70 (20.10, 29.20)	40.20 (26.66, 62.25)	< 0.0001
**Outcomes**				
Hospital stay	9.00 (7.00, 12.00)	10.00 (8.00, 12.00)	3.00 (2.00, 4.00)	< 0.0001
ICU transfer	21 (2.60%)	12 (1.68%)	9 (9.38%)	< 0.0001

Abbreviations and normal range: SFTS, severe fever with thrombocytopenia syndrome; COPD, chronic obstructive pulmonary disease; CHB, chronic hepatitis B; ICU, intensive care unit; Calcium, 8.12-10.16 mg/dL(2.03-2.54mmol/L); Albumin, 35–55 g/L; Corrected calcium, 8.5–10.2 mg/dL; Viral load, < 100TCID50/ml; WBC, white blood cell, 3.5–9.5 × 10^9^/L; RBC, red blood cell, 3.8–5.1 × 10^12^/L; Hb, hemoglobin, 115–150 g/L; PLT, platelet,125–350 × 10^9^/L; ALT, alanine aminotransferase, 5-35U/L; AST, aspartate aminotransferase, 8-40U/L; TBIL, total bilirubin, 5.1-19umol/L; TP, total protein, 64–83 g/L; Globulin, 20–30 g/L; Creatinine, 44-106umol/L; BUN, blood urea nitrogen, 2.9-8.2mmol/L; LDH, lactate dehydrogenase, 109-245U/L; CK, Creatine kinase, 38-174U/L; cTnI, cardiac troponin I, < 26.2ng/L; CKMB, Creatine Kinase MB, < 6.6ng/L; Sodium, 136-145mmol/L; Potassium, 3.5-5.2mmol/L, Magnesium, 0.7-1.1mmol/L; Phosphorus, 0.96-1.62mmol/L; APTT, activated partial thromboplastin time, 28–43.5 s; D-dimer, <0.5ug/mL FEU; FEU, fibrinogen equivalent unit; FIB, fibrinogen, 2.0–4.0 g/L; INR, international normalized ratio, 0.8–1.2; PT, prothrombin time, 11–14.2 s; TT, thrombin time, 14–21 s

Laboratory findings revealed that non-survivors had significantly lower levels of serum albumin, calcium, corrected calcium, platelet count (PLT), lymphocyte count, and monocyte count compared to survivors (all *p* < 0.05). Conversely, non-survivors showed significantly higher levels of viral load; liver function biomarkers (alanine aminotransferase [ALT], aspartate aminotransferase [AST]); renal function biomarkers (creatinine, blood urea nitrogen [BUN]); myocardial injury-related biomarkers (creatine kinase, lactate dehydrogenase [LDH], cTnI, creatine kinase-MB [CK-MB]); coagulation parameters (APTT, D-dimer, international normalized ratio [INR], prothrombin time [PT], thrombin time [TT]); and potassium (all *p* < 0.05).

Among the 809 patients with SFTS, lymphocyte subsets (sTable1) were analyzed in 78 cases (9.6%; survival group: *n* = 71, non-survival group: *n* = 7), and cytokine profiles (sTable2) were assessed in 239 cases (29.5%; survival group: *n* = 214, non-survival group: *n* = 25). Comparative analysis revealed significant immunological differences between the two groups. The survival group exhibited higher absolute counts of B lymphocytes (B cells), CD3 + T cells, CD4 + T cells, and CD8 + T cells compared to the non-survival group. In contrast, natural killer (NK) cell counts were lower in survivors. The most pronounced differences were observed in CD3^+^ T cells, CD4^+^ T cells, and CD8^+^ T cell populations (*P* < 0.05 for all). Non-survivors demonstrated significantly elevated levels of pro-inflammatory and anti-inflammatory cytokines, including IFN-γ, IL-10, IL-4, and IL-6. Conversely, TNF-α and IL-2 levels were lower in non-survivors. The most marked differences were observed in IFN-γ, IL-10, and IL-6 (*P* < 0.05).

### Missing data analysis

Among the 809 patients, 424 (52.4%) had complete data for all laboratory variables, and 385 (47.6%) had at least one missing secondary laboratory variable. As shown in sTable3, the highest missing proportion was observed for CK-MB (25.22%), followed by phosphorus (16.69%) and troponin I (15.70%). Serum calcium, corrected calcium, demographic characteristics, clinical symptoms, and in-hospital mortality had no missing values.

Comparison between the complete-case group and the missing group (sTable4) revealed significant differences in in-hospital mortality, neurological symptoms, hypertension, diabetes, white blood cell count, AST, LDH, creatinine, BUN, and sodium levels (all *P* < 0.05). These differences reflected clinical decision-making: patients with more severe illness or comorbidities received more extensive laboratory workups. Therefore, the missing data were judged to be MAR.

### The association between serum calcium and clinical outcomes

Univariate and multivariate logistic regression analysis on the risk factors associated with mortality of SFTS (Table 2) demonstrated that lower serum calcium as an independent predictor for in-hospital mortality, along with older age, neurological symptoms, higher viral loads, lower albumin and elevated creatinine. Table 3 summarizes the baseline characteristics of all patients stratified by calcium levels. The cohorts were divided into three groups based on baseline corrected calcium levels: G1 <7.0 mg/dL, 7.0 mg/dL ≤ G2<8.12 mg/dL, G3 ≥ 8.12 mg/dL, comprising 59, 529, and 221 patients, respectively. Patients with lower calcium levels were predominantly older, male and exhibited higher rates of neurological symptoms, hemorrhagic signs, ICU admission, and mortality. No significant intergroup differences were observed in comorbidities such as hypertension, diabetes, COPD, and CHB. Mortality, viral load, renal function biomarkers (creatinine and blood urea nitrogen), and coagulation parameters (APTT, D-dimer, fibrinogen [FIB], INR, and PT) were negatively correlated with calcium levels (all *p* < 0.05). In contrast, lymphocyte and monocyte counts were positively correlated with calcium levels (*p* < 0.05).

**Table 2:** Univariate and multivariate logistic regression analysis on the risk factors associated with mortality of SFTS

	Univariate			Multivariate	
	OR (95%CI)	*P*		OR (95%CI)	*P*
Calcium (mg/dL)	0.23 (0.15 ~ 0.35)	< 0.001		0.26 (0.10 ~ 0.66)	0.005
Age	1.09 (1.06 ~ 1.12)	< 0.001		1.13 (1.06 ~ 1.20)	< 0.001
Neurological symptoms	7.80 (4.91 ~ 12.41)	< 0.001		7.91 (3.21 ~ 19.48)	< 0.001
Viral load (Log10)	3.50 (2.73 ~ 4.49)	< 0.001		2.24 (1.44 ~ 3.49)	< 0.001
Albumin	0.93 (0.88 ~ 0.97)	0.002		1.18 (1.03 ~ 1.35)	0.017
Creatinine	1.02 (1.01 ~ 1.02)	< 0.001		1.02 (1.01 ~ 1.02)	< 0.001
APTT	1.06 (1.05 ~ 1.07)	< 0.001		1.04 (1.01 ~ 1.08)	0.033
Hemorrhagic signs	2.05 (1.19 ~ 3.54)	0.01		0.58 (0.14 ~ 2.33)	0.444
Hypertension	2.53 (1.62 ~ 3.94)	< 0.001		1.33 (0.49 ~ 3.58)	0.571
PLT	0.97 (0.95 ~ 0.98)	< 0.001		0.97 (0.95 ~ 1.00)	0.075
Monocyte	0.22 (0.08 ~ 0.64)	0.005		0.61 (0.15 ~ 2.44)	0.48
AST	1.01 (1.01 ~ 1.01)	< 0.001		1.00 (1.00 ~ 1.00)	0.959
LDH(Log10)	31.14 (13.48 ~ 71.94)	< 0.001		1.98 (0.11 ~ 34.59)	0.639
CK(Log10)	2.56 (1.71 ~ 3.82)	< 0.001		0.45 (0.12 ~ 1.67)	0.233
cTnI	1.01 (1.01 ~ 1.01)	< 0.001		1.00 (1.00 ~ 1.00)	0.229
CK-MB	1.05 (1.03 ~ 1.07)	< 0.001		1.01 (0.96 ~ 1.06)	0.693

**Table 3:** Characteristics and outcomes of SFTS patients categorized by calcium on admission

	baseline calcium			*P*
	G1 <7.0 (*n* = 59)	7.0 ≤ G2<8.12 (*n* = 529)	G3 ≥ 8.12 (*n* = 221)	
**Characteristic**				
Age, M (Q₁, Q₃)	66.00 (57.00,71.00)	63.00 (56.00,70.00)	62.00 (54.00,67.00)	0.01
Sex, n(%)	38 (64.41)	216 (40.83)	88 (39.82)	0.002
**Comorbidity**				
Neurological symptoms, n(%)	24 (40.68)	151 (28.54)	41 (18.55)	< 0.001
Hemorrhagic signs, n(%)	14 (23.73)	62 (11.72)	25 (11.31)	0.025
**Complications**				
Hypertension, n(%)	16 (27.12)	125 (23.63)	50 (22.62)	0.77
Diabetes, n(%)	4 (6.78)	67 (12.67)	22 (9.95)	0.284
COPD, n(%)	4 (6.78)	20 (3.78)	9 (4.07)	0.543
CHB, n(%)	5 (8.47)	55 (10.40)	23 (10.41)	0.896
**Laboratory parameters**				
Viral load (Log10)	3.94 (3.11,5.02)	3.16 (2.26,4.11)	2.51 (1.44,3.57)	< 0.001
WBC	2.71 (1.88,4.50)	2.81 (1.70,4.78)	2.95 (1.88,4.91)	0.464
RBC	4.26 (3.82,4.76)	4.16 (3.82,4.57)	4.27 (3.88,4.71)	0.166
Hb	127.00 (113.00,142.00)	125.00 (115.00,139.00)	127.00 (116.00,139.00)	0.539
PLT	40.00 (27.00,53.00)	48.00 (34.00,61.00)	57.00 (39.00,79.00)	< 0.001
Lymphocyte	0.63 (0.41,1.28)	0.64 (0.41,1.08)	0.90 (0.50,1.46)	< 0.001
Monocyte	0.11 (0.06,0.28)	0.15 (0.08,0.33)	0.22 (0.10,0.46)	< 0.001
Neutrophil	1.97 (1.23,2.65)	1.66 (0.93,3.38)	1.59 (0.92,2.95)	0.647
ALT	117.00 (68.00,180.00)	83.00 (49.00,151.00)	67.00 (45.00,113.00)	< 0.001
AST	333.00 (169.00,703.00)	205.00 (109.00,388.00)	138.00 (79.00,231.00)	< 0.001
Tbil	9.50 (7.95,12.50)	9.00 (6.80,12.40)	10.30 (7.80,13.40)	0.005
TP	54.40 (50.70,58.95)	56.25 (52.80,60.02)	60.60 (56.70,64.60)	< 0.001
Albumin	28.60 (25.50,31.80)	31.10 (28.30,33.70)	34.20 (30.90,37.50)	< 0.001
Globulin	25.20 (22.40,28.85)	25.10 (22.50,27.90)	26.05 (23.48,29.30)	0.013
Creatinine	88.85 (68.85,127.50)	68.45 (57.90,85.45)	65.50 (55.95,80.70)	< 0.001
BUN	6.01 (4.49,9.38)	4.81 (3.45,6.50)	4.56 (3.29,6.40)	< 0.001
LDH(Log10)	3.09 (2.89,3.28)	2.89 (2.72,3.12)	2.76 (2.58,2.96)	< 0.001
CK(Log10)	3.12 (2.71,3.44)	2.71 (2.40,3.09)	2.55 (2.16,2.89)	< 0.001
cTnI	107.70 (68.05,419.10)	85.30 (41.50,227.67)	59.30 (21.80,125.20)	< 0.001
CK-MB	6.20 (2.20,12.15)	3.25 (1.60,6.70)	2.80 (1.20,5.40)	< 0.001
Calcium	6.72 (6.56,6.88)	7.64 (7.44,7.88)	8.36 (8.20,8.60)	< 0.001
Sodium	135.40 (131.70,137.95)	135.20 (132.60,137.70)	137.10 (134.20,140.20)	< 0.001
Potassium	3.78 (3.28,4.11)	3.60 (3.29,3.93)	3.67 (3.37,3.96)	0.127
Magnesium	0.71 (0.65,0.80)	0.76 (0.67,0.84)	0.80 (0.73,0.89)	< 0.001
Phosphorus	0.99 (0.77,1.12)	0.87 (0.73,1.04)	0.93 (0.78,1.12)	0.002
APTT	63.50 (50.95,81.43)	51.10 (44.60,60.70)	43.35 (37.88,51.78)	< 0.001
D-dimer	4.17 (2.92,8.62)	2.71 (1.45,5.21)	1.56 (0.91,3.38)	< 0.001
FIB	2.20 (1.71,2.54)	2.47 (2.05,2.89)	2.73 (2.22,3.10)	< 0.001
INR	1.11 (1.03,1.23)	1.02 (0.94,1.12)	1.00 (0.93,1.10)	< 0.001
PT	14.10 (13.20,15.30)	13.10 (12.40,14.20)	12.80 (12.20,13.90)	< 0.001
TT	37.40 (22.70,59.00)	24.60 (20.60,31.40)	21.80 (18.83,26.90)	< 0.001
**Outcomes**				
Fatality, n(%)	22 (37.29)	63 (11.91)	11 (4.98)	< 0.001
Hospitalstay	9.00 (3.50,13.00)	10.00 (7.00,12.00)	9.00 (6.00,11.00)	0.02
ICUtransfer, n(%)	5 (8.47)	14 (2.65)	2 (0.90)	0.005

G1, comprising 59 cases, was associated with significantly higher mortality (37.29%) and an increased likelihood of ICU transfer (8.47%). This group also exhibited the highest levels of viral load; liver function biomarkers (ALT, AST, and total bilirubin [Tbil]); renal function biomarkers (creatinine and BUN); myocardial injury-related biomarkers (LDH, creatine kinase, and CK-MB); and coagulation function biomarkers (APTT, D-dimer, INR, PT, and TT) (all *p* < 0.05), the lowest level of PLT, lymphocyte, monocyte, albumin and magnesium (all *p* < 0.05). Patients in G3 exhibited the lowest mortality rate (4.98%) and ICU transfer rate (0.9%), had the mildest clinical conditions, characterized by the highest lymphocyte and monocyte counts, the lowest levels of viral load, the lowest levels of renal function biomarkers (creatinine and BUN) and coagulation function biomarkers (APTT, D-dimer, INR, PT, and TT) (all *p* < 0.05).

Kaplan-Meier survival analysis results are presented in S1 Fig, demonstrating significant divergence in survival probabilities among the three groups (*p* < 0.001). Hypocalcemia was significantly associated with an increased risk of 28-day all-cause mortality. The highest survival rate was observed in G3, while the lowest survival rate was found in G1.

**Fig. 1:**
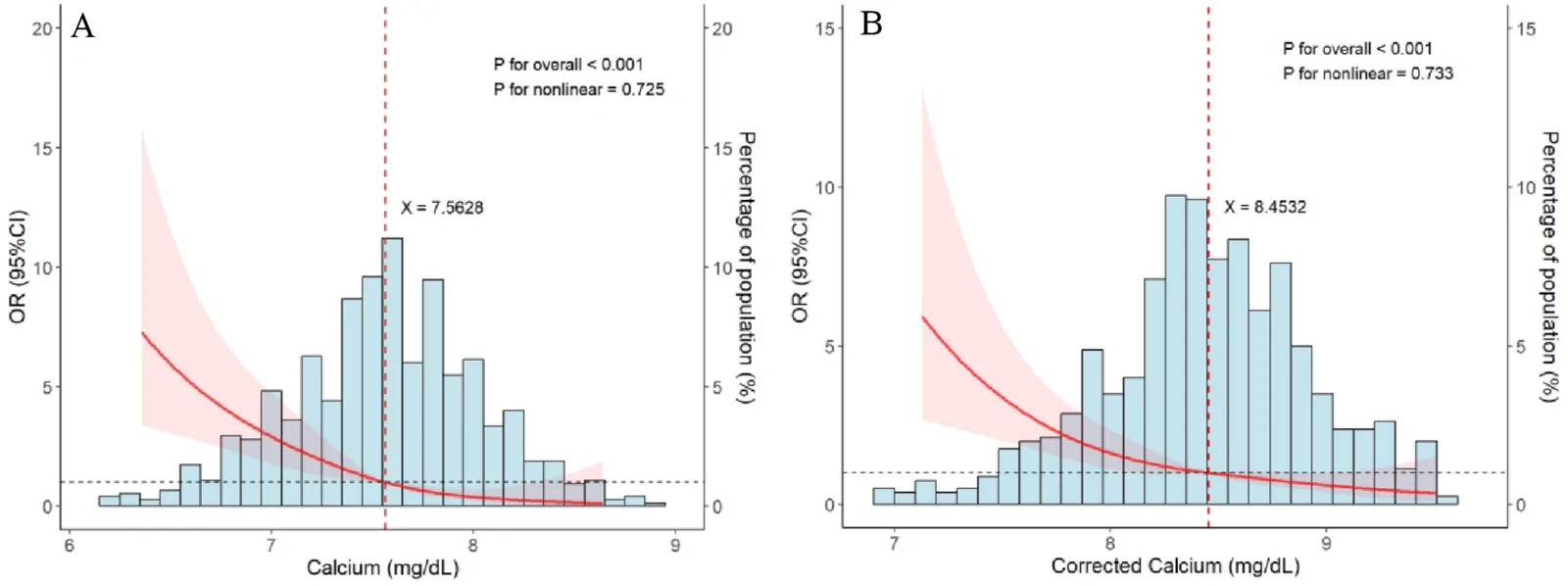
Restricted cubic spline models depicting the relationship between calcium levels and mortality risk in SFTS. **A**, calcium upon admission; **B**, corrected calcium value upon admission. The relative frequency of corrected calcium/calcium is represnted by the blue bars. The 95% CI of the adjusted odds ratio (OR) is represented by the red-shaded areas

As shown in Fig. 1A, RCS analysis revealed a visually L-shaped pattern between serum calcium levels and mortality risk in SFTS patients, with an inflection point around 7.56 mg/dL. Below this point, mortality risk rose with decreasing calcium. The corresponding p-value for non-linearity was 0.733, indicating no statistically significant deviation from linearity. Similar results were observed for albumin-corrected calcium in Fig. 1B (inflection point at 8.4532 mg/dL; *p* = 0.725). The distribution of serum calcium and corrected calcium values in the study population is represented by the histograms, showing that the majority of patients had calcium levels near or below the identified inflection points.

### Dynamic change of serum calcium during hospitalization

**Fig. 2:**
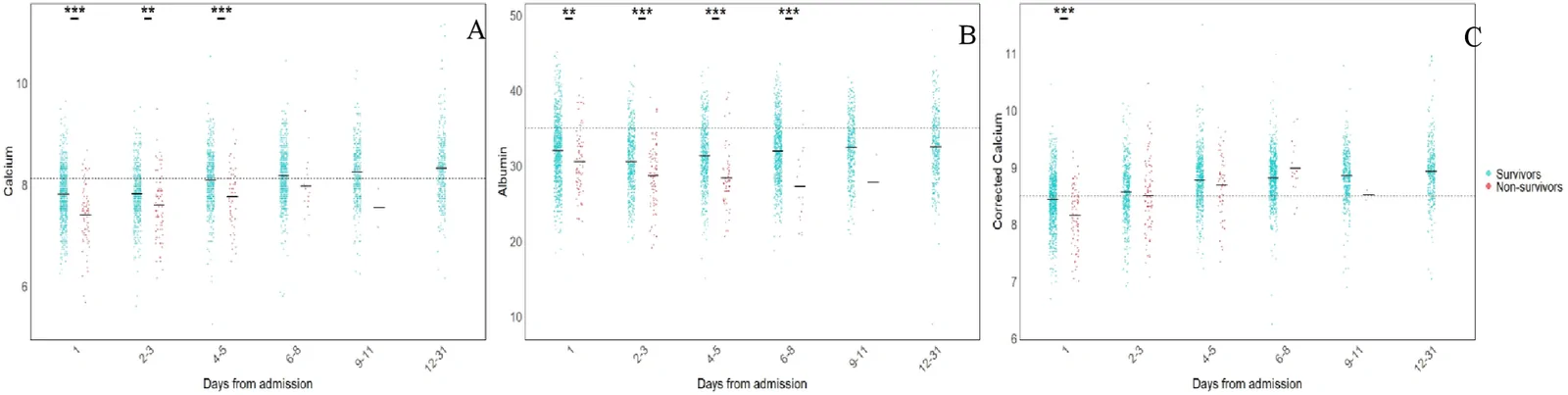
Kinetics of calcium, albumin, corrected calcium between the survivors and non-survivors during hospitalization. **A**, calcium; **B**, albumin; **C**, corrected calcium. All parameters were analyzed at different time intervals for the entire hospital stay. The black solid line represents median of reference parameters, The LLNof reference parameters were depicted with a black dotted line. Three asterisk (***) indicates p <0.001, two asterisks (**) indicate p <0.01, one asterisks (*) indicate p <0.05. LLN=lower limit of normal

**Fig. 3:**
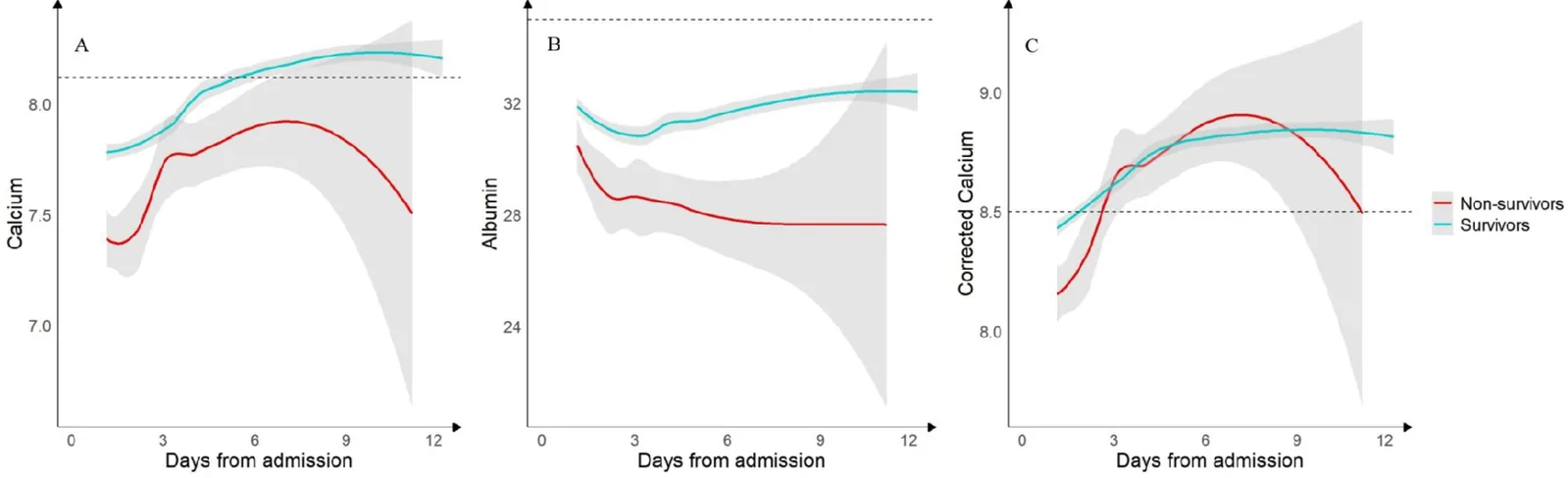
Dynamic profile of calcium, albumin, and corrected calcium between survivors and non-survivors in patients with SFTS. **A**, calcium; **B**, albumin, **C**, corrected calcium. All parameters were analyzed at different time intervals for the entire hospital stay. The LLNof reference parameters were depicted with a black dotted line. LLN, lower level of normal

We conducted a kinetic analysis of calcium, albumin, and corrected calcium levels, tracking their changes from day 1 to day 28 or until discharge after admission (see Fig. 2). The trajectories of calcium, albumin, and corrected calcium levels from day 1 to day 12 or until discharge after admission are shown in Fig. 3. At each time point, the calcium and albumin levels in the survival group were consistently higher than those in the non-survival group. Except for the 6-8th days after admission, the corrected calcium levels in the survival group were consistently higher than those in the non-survival group at all other time points. In the survival group, serum calcium showed an upward trend and reached the normal range by the 6th day after admission. While it gradually increased, reaching its peak on the 6th day and then gradually decreased in the non-survival group.

### Potential factors associated with low serum calcium

**Fig. 4:**
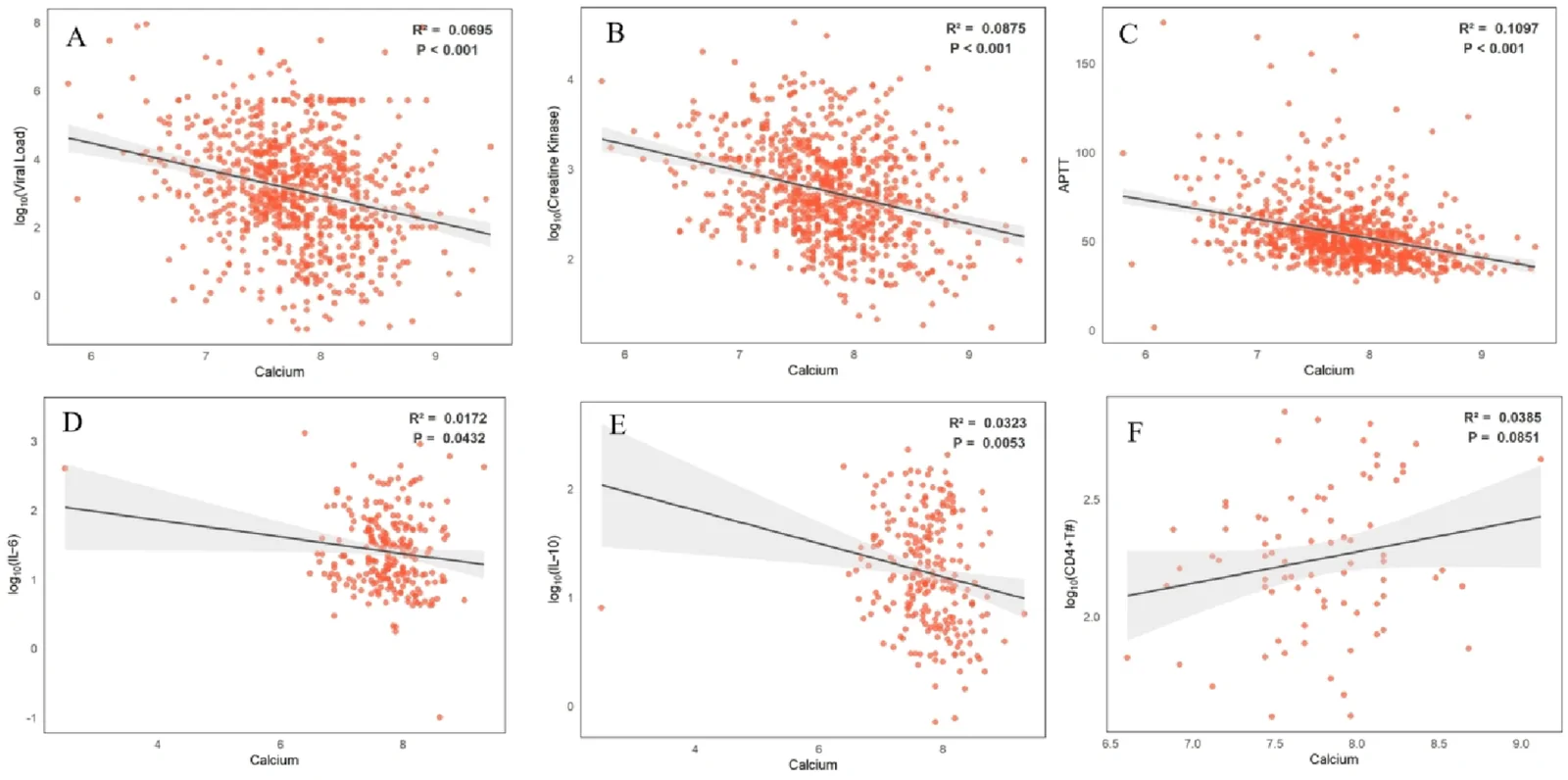
Low calcium levels are associated with high viremia, myocardial damage, coagulation disorder, cytokine storm and T-cell immunodeficiency in patients with SFTS. Linear correlation between calcium and viral load (**A**), creatine kinase (**B**), APTT (**C**), IL-6 (**D**), IL-10 (**E**), CD4+T cell (**F**) in serum

Univariate and multivariate logistic regression analyses of factors associated with hypocalcemia are presented in Table 4. To further investigate the characteristics associated with hypocalcemia in SFTS patients, we stratified the cohort by admission serum calcium levels into hypocalcemia group (calcium < 8.12 mg/dL) and normocalcemia group (calcium ≥ 8.12 mg/dL). Patients with hypocalcemia were significantly older and exhibited higher viral loads, elevated levels of AST, creatinine, BUN, LDH, CK, CK-MB, and prolonged APTT. Conversely, the hypocalcemia group demonstrated significantly lower PLT, lymphocyte and monocyte counts, TP, albumin, magnesium, and sodium levels. Multivariate logistic regression analysis identified several independent factors significantly associated with hypocalcemia at admission. These included lower albumin levels (OR = 0.80, *p* < 0.001), lower globulin levels (OR = 0.93, *p* = 0.002), lower sodium levels (OR = 0.93, *p* = 0.002), and lower monocyte counts (OR = 0.48, *p* = 0.05). These findings indicate that hypoalbuminemia, hypoglobulinemia, hyponatremia, and monocytopenia represent independent risk factors for hypocalcemia in SFTS patients. Linear regression analysis (Fig. 4) revealed statistically significant inverse associations between serum calcium levels and viral loads (R² = 0.0695, *p* < 0.001), CK (R² = 0.0875, *p* < 0.001), APTT (R² = 0.1097, *p* < 0.001), IL-6 (R² = 0.0172, *p* < 0.0432), IL-10 (R² = 0.0323, *p* < 0.0053). However, the R² values were consistently low (ranging from 0.017 to 0.110), indicating that serum calcium levels explain only a small fraction of the variability in these parameters. These associations, while statistically significant in a large sample, should not be overinterpreted as strong evidence of biologically meaningful relationships. These findings suggest that hypocalcemia was independently associated with higher viral load, myocardial injury (indicated by CK elevation), coagulation dysfunction (indicated by prolonged APTT), and cytokine storm (reflected by elevated IL-6 and IL-10 levels) in patients with SFTS.

**Table 4:** Univariate and multivariate logistic regression analysis of risk factors associated with hypocalcemia

	Univariate			Multivariate	
	OR (95%CI)	*P*		OR (95%CI)	*P*
Albumin	0.82 (0.79 ~ 0.86)	< 0.001		0.80 (0.76 ~ 0.85)	< 0.001
Globulin	0.95 (0.92 ~ 0.98)	0.002		0.93 (0.89 ~ 0.97)	0.002
Sodium	0.90 (0.87 ~ 0.94)	< 0.001		0.93 (0.88 ~ 0.98)	0.003
Monocyte	0.42 (0.26 ~ 0.66)	< 0.001		0.48 (0.23 ~ 0.99)	0.050
Age	1.02 (1.01 ~ 1.04)	0.007		1.00 (0.98 ~ 1.02)	0.810
Viral load(Log10)	1.34 (1.20 ~ 1.49)	< 0.001		1.12 (0.95 ~ 1.32)	0.177
PLT	0.99 (0.99 ~ 0.99)	< 0.001		1.01 (1.00 ~ 1.01)	0.139
Lymphocyte	0.65 (0.52 ~ 0.82)	< 0.001		0.89 (0.60 ~ 1.32)	0.568
AST	1.01 (1.01 ~ 1.01)	< 0.001		1.00 (1.00 ~ 1.00)	0.513
Creatinine	1.01 (1.01 ~ 1.01)	0.006		1.01 (1.00 ~ 1.02)	0.121
BUN	1.07 (1.01 ~ 1.13)	0.012		0.98 (0.88 ~ 1.09)	0.715
CK(Log10)	2.40 (1.76 ~ 3.29)	< 0.001		1.08 (0.71 ~ 1.66)	0.719
Magnesium	0.10 (0.03 ~ 0.29)	< 0.001		0.34 (0.08 ~ 1.40)	0.137
APTT	1.04 (1.03 ~ 1.06)	< 0.001		1.01 (0.99 ~ 1.02)	0.514

Abbreviations: CI, confidence interval; OR, odds ratios;

## Discussion

This study explored the relationship between serum calcium levels, their dynamic changes, and in-hospital mortality in patients with SFTS. Our results demonstrate that hypocalcemia is prevalent among SFTS patients upon admission and is significantly associated with the development of neurological symptoms. Furthermore, hypocalcemia emerged as an independent predictor of mortality, alongside other well-established risk factors such as older age, neurological symptoms, higher viral loads, lower albumin, elevated creatinine, and prolonged APTT. These findings suggest that serum calcium may serve as a useful marker for risk stratification in hospitalized SFTS patients.

Our study enrolled 809 SFTS patients, with a mortality rate of 11.87%, consistent with previous reports that mortality in SFTS ranges from 10% to 30% [25]. Hypocalcemia was observed in 72.68% of patients upon admission, indicating its high prevalence in SFTS. This finding aligns with studies on COVID-19, SARS and Ebola viruses, which have also identified hypocalcemia as a common feature in critically ill patients [11, 17, 19]. Hypocalcemia was observed in 60–75% of COVID-19 patients, 60% of SARS patients, and a similar proportion of Ebola virus disease patients [26–28], which was similar with our study. Currently, there is very limited research on serum calcium in SFTS. Only one retrospective study from China found that serum calcium levels were an independent protective factor against in-hospital mortality in SFTS patients [29]. Our study found that calcium levels were strongly associated with the development of neurological symptoms and mortality. Although the mechanisms remain unclear, this association may be related to calcium’s role in cellular signaling, muscle contraction, and coagulation [30]. The RCS analysis visually suggested an L-shaped pattern. However, the test for non-linearity was not statistically significant. Thus, the relationship should be interpreted as primarily linear within our cohort. This finding aligns with previous studies reporting a linear inverse association between calcium levels and mortality in acute myocardial infarction [31]. The dynamic analysis further supported this observation, showing that serum calcium levels in the survival group consistently increased and normalized by the 6th day after admission, whereas in the non-survival group, calcium levels peaked on the 6th day and then declined. Thus, serum calcium may serve as a useful marker for risk stratification in hospitalized SFTS patients. However, the retrospective, single‑centre design of this study limits the generalisability of our findings to other populations or settings. ”

Hypocalcemia is also seen in several viral infections, such as Ebola virus, dengue fever, and COVID-19 [17, 28, 32]. The potential causes of hypocalcemia in SFTS patients may include the following [8, 33–36]: (a) Most SFTS patients experience severe anorexia, nausea, vomiting, and diarrhea, leading to reduced calcium intake and increased excretion; (b) Calcium is primarily bound to plasma albumin, and decreased serum albumin levels can contribute to hypocalcemia; (c) The SFTS virus may directly damage cells, disrupting calcium ion metabolism; (d) Excessive immune and inflammatory responses may cause calcium metabolism disorders, resulting in hypocalcemia; (e) The virus may impair kidney function, affecting calcium reabsorption and vitamin D activation; (f) Medications used in treatment, such as antivirals or antibiotics, may interfere with calcium metabolism; (g) Viral infection may impact the secretion of parathyroid hormone and vitamin D, leading to abnormal calcium metabolism. The dynamic analysis in our study revealed that serum calcium and albumin levels in non-survivors were consistently lower than those in the survivor group. Serum calcium levels were negatively correlated with viral load and renal function markers (creatinine and BUN) and positively correlated with lymphocyte and monocyte counts. Our findings support the above hypothesis. While hypocalcemia was associated with increased mortality, whether correction of serum calcium improves survival requires prospective investigation. Linear regression analysis further indicated that lower serum calcium levels were associated with higher viral loads, along with elevated creatine kinase, prolonged APTT, and increased IL‑6 and IL‑10 levels. These associations are consistent with the hypothesis that hypocalcemia might correlate with higher viral load, myocardial injury, coagulation dysfunction, and inflammatory responses. However, given the observational design of this study, we cannot determine whether these relationships are causal. The findings should be viewed as hypothesis‑generating rather than as evidence of direct mechanisms. Calcium is known to play important roles in cellular homeostasis. It is possible that calcium deficiency could influence viral replication, cardiac function, coagulation, or neurological status, but our observational data do not demonstrate such effects directly [37–40]. These observations are consistent with the known pathophysiology of SFTS, which involves endothelial damage, immune dysregulation, and multi-organ dysfunction [41]. Nevertheless, hypocalcemia is best interpreted as a marker of disease severity rather than a proven causal factor. Additionally, the interplay between calcium levels and other biomarkers, such as viral load, cardiac troponin I, APTT and IL-6 warrants further investigation to better understand the complex pathophysiology of SFTS. It is also worth noting that the R² values for these correlations were low (all < 0.11), suggesting that serum calcium levels account for only a limited proportion of the variation in viral load, inflammatory cytokines, or coagulation parameters. Statistical significance in this large cohort does not necessarily imply clinical or biological importance. These findings should therefore be interpreted cautiously. With this in mind, we next examined the clinical implications of hypocalcemia in our cohort.

Our study found that 72.68% of SFTS patients exhibited hypocalcemia upon admission, and lower serum calcium levels were associated with a significantly increased mortality risk. Specifically, when serum calcium levels fell below 7.56 mg/dL or corrected calcium levels dropped below 8.45 mg/dL, mortality rates rose markedly. This observational study cannot determine whether correcting hypocalcemia improves outcomes. Hypocalcemia likely reflects underlying disease severity and systemic dysregulation. A pilot study conducted in Mexico involving a cohort of dengue-infected patients demonstrated that oral supplementation with calcium carbonate (CaCO₃) combined with vitamin D₃ significantly enhanced platelet count, improved clinical outcomes, and reduced the duration of disease [42, 43]. However, in the context of sepsis management, calcium supplementation has been shown to lack clinical efficacy and may pose potential risks to patient outcomes [44]. For SFTS, there is currently no available data regarding the effects of calcium supplementation in patients with hypocalcemia. Recent studies reported that SFTSV infection induces progressive Ca2 + influx, which can be effectively inhibited by benidipine hydrochloride administration. This treatment not only enhances viral clearance and improves clinical outcomes but also leads to a significant reduction in case fatality rates [11, 37]. Research findings on COVID-19 further corroborated this observation, showing that therapeutic intervention with amlodipine besylate, a calcium channel blocker, led to a decreased case fatality rate among patients [45]. Therefore, future research should prioritize elucidating the pathophysiological mechanisms underlying the association between hypocalcemia and adverse clinical outcomes in SFTS. While serum calcium serves as a valuable prognostic marker, whether its correction directly reduces mortality requires investigation through prospective, interventional trials. Well-designed prospective studies are warranted to evaluate whether early therapeutic interventions, including calcium supplementation or calcium channel blockers (CCBs), can improve survival rates and reduce disease-related complications in this patient population.

This study has several limitations. First, it is a single-center study, and the findings may not be generalizable to other populations, future multicenter studies are necessary to validate our findings. Second, while the study demonstrates associations between calcium levels and outcomes, it does not establish causality. Mechanistic studies are needed to elucidate the underlying pathways linking hypocalcemia to virus replication, myocardial injury, coagulation dysfunction, and cytokine storm in SFTS. Thirdly, the retrospective design limits serum calcium to a prognostic marker, with no therapeutic implications. Fourth, several clinically relevant variables, including specific antiviral regimens, calcium or vitamin D supplementation, nutritional status, and illness severity scores at admission, were not available. Unmeasured or residual confounding may therefore have biased the observed associations. This should be considered when interpreting the observed associations. Fifth, although we used the Payne formula to correct total calcium for albumin, corrected calcium remains an imperfect surrogate for ionized calcium. Systemic inflammation, acid-base disturbances, and altered protein binding-all common in patients with severe SFTS can affect the accuracy of this correction. Without direct ionized calcium measurements, some misclassification of physiologically active calcium levels is inevitable. Therefore, our findings should be interpreted with this limitation in mind. Sixth, some secondary laboratory variables, such as CK-MB, had missing proportions as high as 25.22%, which may introduce some risk of bias. However, these variables were not the primary exposure or outcome of our study. The core exposure (serum calcium) and outcome (in-hospital mortality) had no missing data. Moreover, all baseline variables that differed between the complete-case and missing groups were included in the multiple imputation and regression models to adjust for potential imbalances. Therefore, the impact of missing data on our main conclusions is likely limited.

Declarations:

## Supplementary Information

Below is the link to the electronic supplementary material.

Supplementary Material 1

## Data Availability

The original data generated in this analysis are available from the corresponding author on reasonable request.
