# Supplementary material for: Association between serum calcium and in-hospital mortality in patients with severe fever with thrombocytopenia syndrome: a retrospective cohort study
Source: Virol J. 2026 Jul 7;23:228. doi: 10.1186/s12985-026-03232-z (PMC13625423; doi:10.1186/s12985-026-03232-z)
Supplement: Supplementary file 1 [file 12985_2026_3232_MOESM1_ESM.docx]

**sTable1 Comparison of Lymphocyte Subsets Between Survivors and non-Survivors**

| **Variables** | **Total (n = 78)** | **Survivors (n = 71)** | **Non- survivors (n = 7)** | **P** |
| --- | --- | --- | --- | --- |
| B | 87.12 (49.96, 133.25) | 87.15 (51.43, 149.50) | 66.50 (46.79, 106.93) | 0.576 |
| CD3+T | 381.03 (230.59, 656.97) | 406.10 (231.75, 689.80) | 290.67 (129.75, 320.29) | 0.029 |
| CD4+T | 170.75 (114.31, 290.42) | 180.63 (124.46, 311.87) | 112.95 (82.72, 147.38) | 0.03 |
| CD8+T | 177.85 (76.53, 305.30) | 187.15 (80.65, 307.31) | 121.68 (40.88, 142.57) | 0.043 |
| NK# | 77.61 (40.53, 120.58) | 68.01 (40.12, 118.81) | 82.25 (73.58, 206.67) | 0.215 |

| **Variables** | **Total (n = 239)** | **Survivors (n = 214)** | **Non- survivors (n = 25)** | **P** |
| --- | --- | --- | --- | --- |
| IFN-γ | 4.28 (2.45, 8.59) | 4.00 (2.41, 7.58) | 8.61 (3.11, 21.80) | 0.004 |
| TNF-α | 3.30 (1.85, 6.61) | 3.47 (1.83, 7.08) | 2.76 (1.90, 3.90) | 0.253 |
| IL-10 | 15.75 (6.93, 42.43) | 13.87 (6.62, 35.60) | 64.44 (29.23, 111.81) | <.001 |
| IL-2 | 2.09 (1.48, 3.21) | 2.09 (1.48, 3.24) | 1.97 (1.49, 2.90) | 0.932 |
| IL-4 | 1.88 (1.32, 2.82) | 1.85 (1.29, 2.86) | 2.06 (1.65, 2.51) | 0.568 |
| IL-6 | 22.01 (10.06, 55.32) | 18.93 (9.67, 50.90) | 55.32 (29.66, 203.82) | <.001 |

**sTable2 Comparison of Cytokine Levels Between Survivors and non-Survivors**

**sTable3 Proportion of missing data for each laboratory variable**

| Variable | Missing (n) | Missing (%) |
| --- | --- | --- |
| CK-MB | 204 | 25.22 |
| Phosphorus | 135 | 16.69 |
| cTni | 127 | 15.7 |
| D-dimer | 68 | 8.41 |
| Creatinine | 40 | 4.94 |
| Viral_load | 29 | 3.58 |
| TT | 29 | 3.58 |
| INR | 28 | 3.46 |
| PT | 28 | 3.46 |
| APTT | 26 | 3.21 |
| FIB | 26 | 3.21 |
| LDH | 19 | 2.35 |
| Creatine_kinase | 19 | 2.35 |
| Globulin | 2 | 0.25 |
| Neutrophil | 1 | 0.12 |
| TP | 1 | 0.12 |
| BUN | 1 | 0.12 |
| Magnesium | 1 | 0.12 |
| Group | 0 | 0 |
| Fatality | 0 | 0 |
| Calcium | 0 | 0 |
| Corrected Calcium(mg/dL) | 0 | 0 |
| Sex | 0 | 0 |
| Age | 0 | 0 |
| Neurological_symptoms | 0 | 0 |
| Bleeding | 0 | 0 |
| Hypertension | 0 | 0 |
| Diabetes | 0 | 0 |
| COPD | 0 | 0 |
| CHB | 0 | 0 |
| ICU_transfer | 0 | 0 |
| Hospital_stay | 0 | 0 |
| WBC | 0 | 0 |
| RBC | 0 | 0 |
| Hb | 0 | 0 |
| PLT | 0 | 0 |
| Lymphocyte | 0 | 0 |
| Monocyte | 0 | 0 |
| ALT | 0 | 0 |
| AST | 0 | 0 |
| Tbil | 0 | 0 |
| Albumin | 0 | 0 |
| Sodium | 0 | 0 |
| Potassium | 0 | 0 |

**sTable4 Comparison of baseline characteristics between complete-case and missing groups**

| Variable | Complete-case (n=424) | Missing (n=385) | P value |
| --- | --- | --- | --- |
| Sex, n (%) | 185 (43.6) | 157 (40.8) | 0.4538 |
| Fatality, n (%) | 61 (14.4) | 35 (9.1) | 0.0266 |
| Group, n (%) | 100 (23.6) | 97 (25.2) | 0.6538 |
| Neurological symptoms, n (%) | 128 (30.2) | 88 (22.9) | 0.0229 |
| Bleeding, n (%) | 53 (12.5) | 48 (12.5) | 1 |
| Hypertension, n (%) | 121 (28.5) | 70 (18.2) | 0.0007 |
| Diabetes, n (%) | 35 (8.3) | 58 (15.1) | 0.0035 |
| COPD, n (%) | 21 (5.0) | 12 (3.1) | 0.2541 |
| CHB, n (%) | 38 (9.0) | 45 (11.7) | 0.246 |
| ICU transfer, n (%) | 10 (2.4) | 11 (2.9) | 0.8227 |
| Age, median (IQR) | 63 (55, 70) | 62 (55, 69) | 0.2002 |
| WBC, median (IQR) | 2.55 (1.73, 4.11) | 3.28 (1.84, 5.48) | 0.0001 |
| PLT, median (IQR) | 50 (33.75, 61) | 49 (35, 70) | 0.0522 |
| ALT, median (IQR) | 82 (47.75, 146.5) | 79 (49, 140) | 0.9629 |
| AST, median (IQR) | 202 (104.75, 393.25) | 176 (97, 318) | 0.0463 |
| LDH, median (IQR) | 790 (525.5, 1313.5) | 690 (452.5, 1145.5) | 0.0045 |
| Albumin, median (IQR) | 31.4 (28.3, 34.5) | 31.9 (29.2, 34.8) | 0.1351 |
| Creatinine, median (IQR) | 70.05 (58.8, 90.05) | 66.2 (57.1, 83.5) | 0.0157 |
| BUN, median (IQR) | 4.93 (3.57, 7.03) | 4.7 (3.36, 6.3) | 0.0219 |
| Sodium, median (IQR) | 134.95 (132, 137.4) | 136.5 (133.8, 139.4) | P<0.0001 |
| Potassium, median (IQR) | 3.63 (3.26, 3.94) | 3.63 (3.36, 3.96) | 0.6549 |

**S1 Fig Flowchart of participants enrollment in the study**

**S2 Fig Kaplan-Meier survival analysis curves according to the serum calcium levels between the three groups**
